# Matrix-assisted laser desorption/ionization time-of-flight mass spectrometry: A breakthrough in rapid and precise mold identification

**DOI:** 10.22034/cmm.2025.345248.1617

**Published:** 2025-08-26

**Authors:** Aparna Naik, Kanchan Ajbani, Asmita Salvi, Camilla Rodrigues, Shaoli Basu, Anjali Shetty

**Affiliations:** 1 Department of Microbiology, P. D. Hinduja Hospital and Medical Research Center, Mumbai, India

**Keywords:** MALDI TOF-MS, *Aspergillus*, *Fusarium*, Rhizopus Mold identification

## Abstract

**Background and Purpose:**

Rapid and accurate identification of molds is critical in clinical microbiology, particularly for immunocompromised patients at increased risk of fungal infections. Traditional methods, such as culture and microscopy, are time-consuming and may lack species-level specificity. The matrix-assisted laser desorption/ionization time-of-flight mass spectrometry matrix-assisted laser desorption/ionization time-of-flight mass spectrometry (MALDI-TOF MS) provides a promising alternative due to its speed and accuracy. This study aimed to standardize Vitek MS for mold identification and compare its effectiveness with traditional phenotypic methods, assessing its reliability, turnaround times, and cost-effectiveness in clinical settings.

**Materials and Methods:**

A retrospective examination of 248 anonymized mold isolates was conducted at Hinduja Hospital, with 182 isolates successfully revived. Both Vitek MS and phenotypic techniques were used to identify the molds, and the concordance rate between the two methods was calculated.

**Results:**

Vitek MS accurately identified 169 out of the 182 isolates, yielding a concordance rate of 92.85% with phenotypic methods. Of the remaining isolates, 1 was misidentified, 4 could not be identified, and 8 were not represented in the database.

**Conclusion:**

Vitek MS proved to be a highly accurate and time-efficient tool for mold identification, enhancing clinical diagnostics. Expansion of its database is crucial for broader mold identification. This study supported integration of Vitek MS into routine laboratory practice, aiding in timely antifungal treatment, improving patient outcomes, and assisting in outbreak detection and public health management.

## Introduction

Molds are diverse fungi that can cause infections, particularly in immunocompromised individuals, making accurate identification crucial for effective diagnosis and treatment. Traditional methods, such as culture and microscopy, are time-consuming and often lack species-level specificity. Advent of Matrix-Assisted Laser Desorption/Ionization Time of Flight Mass Spectrometry (MALDI-TOF MS) offered a promising alternative, providing rapid and precise results. Although this technique has revolutionized bacterial identification, its application for molds is still evolving, reliant on comprehensive reference databases and optimized extraction protocols [ 1 , 2 ]. Rapid and accurate mold identification is critical, as molds can lead to life-threatening infections. Correct identification permits prompt antifungal medication, decreasing morbidity and mortality rates, and benefits public health by allowing the early detection of fungal outbreaks [ 3 ]. However, challenges persist, including gaps in reference databases for rare species and the need for optimized extraction protocols [ 4 , 5 ].

This study aimed to standardize Vitek MS for mold identification by developing and validating protocols that ensure high accuracy and reproducibility. In this regard, the performance of Vitek MS was compared with that of traditional methods regarding identification accuracy and turnaround time, ultimately demonstrating its clinical utility in routine laboratory practice [ 6 , 7 ].

## Materials and Methods

### Study design

This study aimed to standardize the Vitek MS technique for mold identification and speciation. Methodologies included optimizing protein extraction protocols, comparing Vitek MS with traditional phenotypic methods, and validating results using American Type Culture Collection (ATCC) isolates. Focus of this study was on enhancing accuracy and reproducibility through optimized protocols [ 8 ] and rigorous testing.

### Sample selection

In this study, 248 anonymized mold isolates were obtained from a biobank at the reference fungal laboratory of the hospital. Successful revival on Sabouraud Dextrose Agar (SDA) or Potato Dextrose Agar (PDA) was the inclusion criterion. Ultimately, 182 isolates were successfully revived and included in the study, while 66 isolates that could not be revived were excluded.

### Revival and culturing

Isolates were revived on SDA and PDA under optimal conditions at 35 °C and room temperature until sporulation occurred. Only those exhibiting healthy growth were used for subsequent analyses [ 9 ].

### Phenotypic methods

Traditional culture methods were employed to grow mold isolates. Key morphological features, such as colony color (obverse and reverse), texture, growth rate, hyphae structure, and spore characteristics, were examined microscopically for classification at the genus or species level. These phenotypic methods served as the standard for comparison with Vitek MS results.

### Vitek MS Identification

Vitek MS was used for species-level identification, employing a standardized protein extraction protocol [ 10 ].

### Protein extraction protocol (Vitek MS mold kit)

Young sporulating colonies were collected using sterile swabs and suspended in 0.9 mL of R1 buffer in a 2 mL tube. After vortexing and centrifuging at 14,000 RPM for 2 min, the supernatant was discarded. The pellet was resuspended in 40 µL of R2, vortexed, mixed with 40 µL of R3, and subjected to centrifugation. A 2 µL aliquot of the supernatant was spotted onto a target slide, air-dried, and layered with 1 µL of VITEK® MS-CHCA matrix [ 11 ].

### Protein extraction protocol (in-house reagents)

Young sporulating colonies were collected with sterile swabs moistened with sterile distilled water and suspended in 70% ethanol. After vortexing and centrifugation, the supernatant was discarded, and 40 µL of 70% formic acid was added to the pellet. Following a resting period, 40 µL of acetonitrile was added before centrifugation. The supernatant was then spotted onto the target slide, air-dried, and layered with 1 µL VITEK® MS-CHCA matrix [ 12 ].

### Data Acquisition and Analysis

The target slide was introduced into the Vitek MS instrument, and mass spectra were acquired based on unique protein profiles. The spectra were analyzed against a reference database for species identification, and a comprehensive comparison with traditional phenotypic methods was conducted to calculate the concordance rate. Morphological characteristics, such as colony appearance, color, and growth rate, were recorded alongside microscopic analyses of spore structures and hyphae [ 13 ].

### Data comparison and validation

Validation involved comparing Vitek MS results with phenotypic identification. The ATCC reference strains of *Aspergillus flavus* (ATCC 204304) and *Aspergillus terreus* (ATCC MYA 3633) were processed 10 times on consecutive days. Proficiency testing strains from 10 previous years were used for verification of the method. This cross-validation ensured the reliability of Vitek MS for mold identification, confirming high concordance rates and highlighting areas for further database expansion [ 14 ]. It should be mentioned that 64 isolates were used to validate the protein extraction protocol using in-house reagents by the Vitek MS mold identification kit.

A confidence value of 90 or higher was considered to be reliable. If a lower score was obtained, then the protein extraction protocol was repeated from a fresh subculture of the isolate on SDA. It was found that protein extraction may be improved by fully homogenizing the isolate (spores and hyphae) in ethanol and appropriately blending the reagents. Furthermore, extraction is more effective when mold colonies are freshly subcultured on SDA or PDA, are in an early growth phase, and have only recently begun to sporulate. An older culture may not produce satisfactory results. To enhance identification, 2 µL of supernatant should be applied to the target slide and air dried, followed by the addition of another 2 µL of supernatant before overlaying it with 1 µL of VITEK® MS-CHCA matrix.

### Statistical Analysis

Concordance calculations determined the percentage of agreement between Vitek MS and phenotypic identification methods. Cohen's Kappa statistic (κ) was applied to evaluate the level of agreement beyond chance. A Kappa value of 0 indicates no agreement, while values closer to 1 indicate strong agreement [ 15 ]. Additionally, sensitivity, specificity, positive predictive value, and negative predictive value were calculated using ATCC isolates as a reference to further assess the accuracy and reliability of Vitek MS.

## Results

As recommended in the M58 CLSI document, verification was achieved by comparing identification results obtained using MALDI-TOF MS. Compared with the phenotypic identification method, 169 (92.85%) out of 182 isolates were identified correctly. Table 1 summarizes the comparison between phenotypic and Vitek MS identification of fungal species. Key highlights include that the in-house reagent method showed 100% concordance with the kit-based method while significantly reducing the cost. The kit-based method costs ₹ 35,000 (407.7 USD) for 100 tests, i.e., ₹ 350 (4 USD) (excluding the instrument and accessories cost) per test. However, the in-house reagents cost about ₹ 8-10 (0.12 USD) per test (excluding the instrument and accessories cost). Hence, it is also a very cost-effective method if the initial instrument cost is not considered. 

**Table 1:** Species-level identification of molds by Vitek MS vs. phenotypic method on anonymized isolates

Phenotypic Identification	Vitek MS identification	No. of isolates verified on Vitek MS	Concordance (%)	Remarks
*Aspergillus flavus (51)*	*Aspergillus flavus/oryzae (49)*	49	100	
	*Aspergillus tamarii (2)*	2	100	Part of the *Aspergillus flavus* group
*Aspergillus fumigatus (18)*	*Aspergillus fumigatus (18)*	18	100	
*Aspergillus nidulans (3)*	*Aspergillus nidulans (3)*	3	100	
*Aspergillus niger (12)*	*Aspergillus niger complex (12)*	12	100	
*Aspergillus species (1)*	*Aspergillus lentulus (1)*	1	100	Identification to the species level was possible
*Aspergillus sydowii (5)*	*Aspergillus sydowii (5)*	5	100	
*Aspergillus terreus (4)*	*Aspergillus terreus complex (4)*	4	100	
*Cladophialophora bantiana (1)*	*Cladophialophora bantiana (1)*	1	100	
*Coccidioides immitis (1)*	*Coccidioides immitis/posadasii (1)*	1	100	
*Curvularia lunata (2)*	*Curvularia lunata (2)*	2	100	
*Epicoccum nigrum (1)*	*Epicoccum nigrum (1)*	1	100	
*Exophiala dermatitidis (2)*	*Exophiala dermatitidis (2)*	2	100	
*Exophiala spinifera (1)*	*Exophiala spinifera (1)*	1	100	
*Fusarium dimerum (3)*	*Fusarium dimerum (3)*	3	100	
*Fusarium species (2)*	*Fusarium oxysporum complex (1)*	1	100	Identification to the species level was possible
	*Fusarium solani complex (1)*	1	100
*Fusarium solani (14)*	*Fusarium solani complex (14)*	14	100	
*Histoplasma capsulatum (1)*	*Histoplasma capsulatum (1)*	1	100	
*Microsporum canis (1)*	*Microsporum canis (1)*	1	100	
*Microsporum gypseum (1)*	*Microsporum gypseum (1)*	1	100	
*Lichteimia corymbifera (1)*	*Lichteimia corymbifera (1)*	1	100	
*Paeciliomyces variotii (1)*	*Paeciliomyces variotii complex (1)*	1	100	
*Penicillium species (1)*	*Penicillium citrinum (1)*	1	100	Identification to the species level was possible
*Purpureocillium lilacinum (1)*	*Purpureocillium lilacinum (1)*	1	100	
*Rhizopus arhizus (21)*	*Rhizopus arhizus complex (18)*	18	85	
	No ID (3)			Identified by Sanger's sequencing
*Rhizopus microporus (7)*	*Rhizopus microporus complex (6)*	6	85	
	No ID(1)			Identified by Sanger's sequencing
*Scedosporium apiospermum (9)*	*S. apiospermum (7)*	7	88	
	*Pseudallescheria boydii/(S. boydii) (1)*	1	100	Previously considered as the Teleomorph of *S. apiospermum*, but now, based on phylogenetic classification, reclassified as *S. boydii*
	*Metarhizum anisopliae var anisopliae (1)*	1		Identification confirmed by the postgraduate institute of medical education and research in Chandigarh as *S. apiospermum*
*Trichophyton interdigitale (2)*	*Trichophyton interdigitale (2)*	2	100	
*Trichophyton rubrum (3)*	*Trichophyton rubrum (3)*	3	100	
*Trichophyton species (2)*	*Trichophyton interdigitale (2)*	2	100	Identification to the species level was possible
*Trichophyton tonsurans (2)*	*Trichophyton tonsurans (2)*	2	100	
*Schizophylum commne*	No ID (3)	0	0	Not available in database
*Syncephalastrum racemosum*	No ID (2)	0	0	Not available in database
*Paecilomyces variotii*	No ID (3)	0	0	Not available in database

Results of Vitek MS were compared with phenotypic identification results. Concordance between the two methods was calculated to evaluate the accuracy of MALDI-TOF MS.

The DNA sequencing was performed on 5 of the 13 discrepant isolates. Four of these yielded no identification by MALDI-TOF MS, despite *Rhizopus* being present in the database. However, their phenotypic features were consistent with *Rhizopus arrhizus* and *Rhizopus microsporus*. Sequencing confirmed the identification as *Rhizopus arrhizus* (n=3) and *Rhizopus microsporus* (n=1).

The fifth isolate was identified by MALDI-TOF MS as Metarhizium anisopliae var. anisopliae, but phenotypically resembled *Scedosporium apiospermum*. Sequencing confirmed it as *S. apiospermum*.

The remaining eight isolates, which were not represented in the MALDI-TOF MS database, were not sent for sequencing but were used to assess whether MALDI-TOF MS would yield any alternative or incorrect identification, thereby providing a limited indication of system specificity.

### Overall concordance

Vitek MS demonstrated a high concordance rate, accurately identifying 169 out of 182 mold isolates, resulting in a concordance rate of 92.85% (169/182) with traditional methods, indicating that Vitek MS reliably identifies the majority of mold species. To validate the accuracy of Vitek MS, 10 ATCC reference isolates were analyzed, achieving a 100% concordance rate by correctly identifying all isolates. This confirmed the reliability and precision of Vitek MS for the identification of known reference strains. It showed good concordance among *Aspergillus* and *Fusarium* species without any reruns.

### Time efficiency

Culture is a prerequisite for phenotypic, MALDI-TOF MS (Vitek MS), and sequencing methods. However, the duration from culture to final identification varies. Phenotypic identification often requires extended incubation, slide cultures, and temperature-specific subcultures, resulting in a turnaround time of 5–10 days or more. The MALDI-TOF MS can be performed directly on primary cultures in 48–72 h, with actual identification taking only a few hours. However, sequencing requires a pure culture followed by DNA extraction, amplification, sequencing, and analysis, generally taking 5–7 days.

To achieve a healthy mold growth on SDA, it may take from 48 h to one week, depending on the development rate of different species. For example, *Rhizopus*, *Aspergillus*, and *Fusarium* will grow in 24–48 hours, while other molds like Trichophyton may take up to a week. Vitek MS greatly decreases identification time to a few hours, including sample preparation and data processing, as opposed to standard phenotypic approaches, which normally take several days to weeks. This short turnaround time improves clinical decision-making. To differentiate among the species in *Fusarium*, an additional 4–5 days may be required to look for the complete septation in the macroconidia for a confirmatory identification, but if Vitek MS is used, these additional incubation periods can be reduced to a few hours, including sample preparation and data analysis, compared to traditional phenotypic methods.

### Database limitations

This study identified limitations related to the Vitek MS reference database, as some mold species were not represented, leading to instances of misidentification or failure to identify certain isolates. Continuous efforts to expand and update the database are essential for improving Vitek MS accuracy.

### Species-level identification

Vitek MS exhibited superior capability in distinguishing closely related mold species within the *Aspergillus*, *Fusarium*, and *Trichophyton* species and Penicillium genera, often achieving identification where phenotypic methods were insufficient. For instance, it successfully differentiated between *Aspergillus flavus* and *Aspergillus tamarii*.

### Cryptic species identification

Vitek MS exhibited superior capability in distinguishing between species that showed minimal or no morphological differences, such as *Aspergillus sydowii*, *Aspergillus lentulus*, and *Aspergillus fumigatus*, as well as between *Trichophyton interdigitale* and *Trichophyton mentagrophyte*.

### Protein extraction protocol

The standardized protein extraction protocol using formic acid and acetonitrile effectively prepared samples for Vitek MS analysis, ensuring reproducibility across isolates and contributing to the observed high concordance rate.

### Phenotypic vs. Vitek MS identification

While both methods demonstrated high concordance, discrepancies were noted in cases where phenotypic characteristics were ambiguous. Vitek MS proved more precise in identifying species that were difficult to distinguish using traditional methods.

### Statistical Analysis

Cohen's Kappa statistic (κ) was calculated to assess agreement between Vitek MS and phenotypic identification methods. A Kappa value of 0.88 indicated substantial agreement between the two methods, underscoring the robustness of Vitek MS in mold identification compared to traditional techniques.

## Discussion

This study highlighted the relevance of MALDI-TOF MS in mold identification, supporting existing research that underscores its potential in clinical microbiology. Previous studies [ 15 , 16 ] have established the efficacy of this technique, demonstrating high concordance with traditional methods. Focus of this study on standardizing MALDI-TOF MS for mold identification contributes to efforts to integrate this technology into routine clinical practice.

While traditional phenotypic methods are considered the gold standard, they are time-consuming and often imprecise, especially for closely related species. Findings of this study align with those of previous studies [ 17 ], confirming that MALDI-TOF MS not only reduces identification time but also enhances accuracy, particularly in distinguishing closely related species.

A key factor influencing MALDI-TOF MS performance is the comprehensiveness of its reference database. Gaps in the database can lead to misidentifications, a challenge noted by other studies [ 6 , 7 ]. This study reinforced the need for continuous database expansion to improve identification accuracy, especially for rare species. As the database grows, the incidence of misidentifications is likely to decrease.

Time efficiency is another major advantage of MALDI-TOF MS. Traditional methods may take days to weeks for results, while MALDI-TOF MS can deliver accurate identifications within hours. This rapid turnaround is crucial for managing invasive fungal infections, supporting findings by other studies [ 4 , 5 ] regarding the clinical benefits of prompt identification. Precision of MALDI-TOF MS in distinguishing closely related species represents a significant advancement over traditional methods. Results of this study confirmed these findings [ 8 , 9 ], demonstrating the ability of this technique to provide reliable identifications essential for targeted treatment.

Standardization of the protein extraction protocol was critical for achieving reproducible results, aligning with previous research [ 10 ]. The present study showed that consistent sample preparation enhanced the reliability of MALDI-TOF MS, making it a dependable tool for routine mold identification. Future research should focus on the following key areas to enhance MALDI-TOF MS utility in fungal diagnostics:

**Database expansion:** Increasing the reference database to encompass a broader range of mold species, especially rare pathogens, to enhance accuracy.

**Longitudinal studies:** Monitoring MALDI-TOF MS performance over time and across various clinical settings to gather data on long-term reliability.

**Integration with clinical workflows:** Developing protocols to incorporate MALDI-TOF MS into routine clinical practices, ensuring proper training for laboratory personnel.

**Comparative studies:** Conducting studies with emerging molecular techniques, such as next-generation sequencing, to evaluate the strengths and limitations of different identification methods.

## Conclusion

The findings reinforced the value of Vitek MS as a rapid, accurate, and reliable tool for mold identification. Addressing database limitations and standardizing protocols can significantly improve fungal diagnostics, enhancing patient care and outcomes. This research supported the growing evidence for adopting Vitek MS in clinical laboratories, emphasizing its potential to transform fungal diagnostics and treatment, streamline workflows, and ensure timely management of fungal infections.
